# The mitochondrial genome of *Parascaris univalens* - implications for a “forgotten” parasite

**DOI:** 10.1186/1756-3305-7-428

**Published:** 2014-09-04

**Authors:** Abdul Jabbar, D Timothy J Littlewood, Namitha Mohandas, Andrew G Briscoe, Peter G Foster, Fritz Müller, Georg von Samson-Himmelstjerna, Aaron R Jex, Robin B Gasser

**Affiliations:** Faculty of Veterinary and Agricultural Sciences, The University of Melbourne, Melbourne, Victoria Australia; Department of Life Sciences, Natural History Museum, Cromwell Road, London, SW7 5BD UK; Department of Biology, Zoology, Chemin du Musée 10, CH-1700 Fribourg, Switzerland; Institute for Parasitology, Tropical Veterinary Medicine, 14163 Berlin, Germany

**Keywords:** *Parascaris univalens* (Nematoda: Ascaridida), Mitochondrial genome, Genetic markers, Epidemiology, Population genetics

## Abstract

**Background:**

*Parascaris univalens* is an ascaridoid nematode of equids. Little is known about its epidemiology and population genetics in domestic and wild horse populations. PCR-based methods are suited to support studies in these areas, provided that reliable genetic markers are used. Recent studies have shown that mitochondrial (mt) genomic markers are applicable in such methods, but no such markers have been defined for *P. univalens*.

**Methods:**

Mt genome regions were amplified from total genomic DNA isolated from *P. univalens* eggs by long-PCR and sequenced using Illumina technology. The mt genome was assembled and annotated using an established bioinformatic pipeline. Amino acid sequences inferred from all protein-encoding genes of the mt genomes were compared with those from other ascaridoid nematodes, and concatenated sequences were subjected to phylogenetic analysis by Bayesian inference.

**Results:**

The circular mt genome was 13,920 bp in length and contained two ribosomal RNA, 12 protein-coding and 22 transfer RNA genes, consistent with those of other ascaridoids. Phylogenetic analysis of the concatenated amino acid sequence data for the 12 mt proteins showed that *P. univalens* was most closely related to *Ascaris lumbricoides* and *A. suum*, to the exclusion of other ascaridoids.

**Conclusions:**

This mt genome representing *P. univalens* now provides a rich source of genetic markers for future studies of the genetics and epidemiology of this parasite and its congener, *P. equorum*. This focus is significant, given that there is no published information on the specific prevalence and distribution of *P. univalens* infection in domestic and wild horse populations.

**Electronic supplementary material:**

The online version of this article (doi:10.1186/1756-3305-7-428) contains supplementary material, which is available to authorized users.

## Background

Parasitic worms of the gastrointestinal tracts of equids cause diseases of major veterinary importance. For instance, *Parascaris* is a large, parasitic nematode of the small intestine and has a direct life cycle [1, 2]. Infective eggs (each containing a third-stage larva, L3) are ingested by the equid, hatch in the intestine, L3s undergo liver and lung (hepato-pulmonary) migration, moult to fourth-stage larvae, are swallowed and then establish in the small intestine where they mature, mate and reproduce; female worms lay millions of eggs, which pass in the faeces into the environment [1, 2]. Infection with large numbers of adult worms, particularly in foals, can cause colic associated with enteritis and/or intestinal impaction/obstruction, weight loss and anorexia [2, 3]. Migrating larval stages can also cause hepatitis and pneumonitis, associated respiratory disorders (mild signs of coughing and nasal discharge) and secondary bacterial infections [2, 3]. Foals are particularly susceptible to infection and are clinically most affected, but immunity usually develops by the age of 6–12 months [2], such that infections are eliminated from older horses, unless there is a problem with immunosuppression or immunodeficiency.

Parascariasis, the disease caused by *Parascaris*, is treated using anthelmintics such as benzimidazoles (fenbendazole, febantel, oxfendazole and oxibendazole), macrocyclic lactones (including ivermectin and moxidectin), piperazine or pyrantel. As a high frequency of anthelmintic treatment is considered to be a key factor for the selection of drug resistance, this approach is not sustainable in relation to drug efficacy. Accordingly, in the past decade resistance against commonly used anthelmintics [4] has been reported in *Parascaris* [5–13], but it is not yet clear which species of *Parascaris* is involved in this resistance.

Presently, two species of *Parascaris* are recognised, namely *P. univalens* and *P. equorum*. Classical, cytological techniques can be used to identify *P. univalens* and distinguish it from *P. equorum*; the former nematode has two chromosomes, whereas the latter has four [14–18]. Chromosomal banding patterns studied during the gonial metaphase have shown that *P. univalens* chromosomes contain only terminal heterochromatin, whereas *P. equorum* chromosomes also contain intercalary heterochromatin [15]. Although chromosomal differences allow their specific identification, in most, if not all, parasitological and epidemiological studies of equine parasites conducted to date, the specific status of *Parascaris* was not verified. The assumption has been that *P. equorum* is the only or the dominant species of *Parascaris*. However, some recent results from a cytological study of *Parascaris* from domestic horses in northern Germany have shown that *P. univalens* has a higher prevalence than previously expected (G. von Samson-Himmelstjerna *et al.,* unpublished findings). Indeed, *P. equorum* was hardly found. This raises questions about the prevalence and clinical relevance of *P. univalens* as well as drug resistance in this species in countries around the world.

While cytological analysis is a useful method for specific identification and differentiation, it would be desirable to have available genomic markers for PCR-based analyses of genetic variation within *Parascaris* (at any stage of development) as well as the specific diagnosis of infection. Recent studies have shown that mitochondrial (mt) genomic markers are suited for this purpose [19–22]. Although mt genomes have been published for numerous ascaridoids, including *A. suum*, *A. lumbricoides*, *Ascaridia columbae*, *As. galli*, *Baylisascaris procyonis*, *B. transfuga, B. ailuri, B. schroederi, Anisakis simplex*, *Contracaecum osculatum, C. rudolphii* B, *Cucullanus robustus*, *Toxocara canis*, *T. cati, T. malaysiensis* and *Toxascaris leonina* [23–33], this is not the case for *Parascaris*. Therefore, defining a mt genome for *P. univalens* could provide a rich source of markers to underpin detailed investigations of the genetic composition of *Parascaris* populations in domestic and wild horses around the world. The aim of the present study was to utilize a next-generation sequencing-based approach for the characterisation of the mt genome of *P. univalens* from Switzerland as a foundation for such future investigations.

## Methods

### Parasite and genomic DNA isolation

In 1999, eggs of *P. univalens* were collected from an adult female specimen of *Parascaris* from the small intestine from a domesticated horse. This horse was slaughtered for meat in an approved abattoir in Fribourg, Switzerland, and the worms were provided to one of the authors by a registered veterinarian. This work was approved under the Scientific Procedures Premises License for the Faculty of Science, University of Fribourg. The specific identity of the worm was based on cytological examination [15] of the eggs taken from the uterus of this female worm. Total genomic DNA was purified from eggs by sodium dodecyl-sulphate/proteinase K treatment, phenol/chloroform extraction and ethanol precipitation and purified over a spin column (Wizard Clean-Up, Promega) [34].

### Long-PCR, sequencing, mt genome assembly and annotation

Using each of the primer pairs MH39F-MH38R and MH5F-MH40R [35], two regions of the entire mt genome (of ~5 and 10 kb, respectively) were amplified by long-range PCR (BD Advantage 2, BD Biosciences) from 50 ng of genomic DNA [35]. The cycling conditions (in a 2720 thermal cycler, Applied Biosystems) were: one cycle at 95°C for 1 min (initial denaturation), followed by 35 cycles of 95°C for 15 s (denaturation), 53°C for 15 s (~5 kb region) or 55 for 15 s (~10 kb region) (annealing) and 62°C for 5 min (~5 kb region) or 68°C for 6 min (~10 kb region) (extension), followed by a final elongation at 62°C or 68°C for 5 min [35]. Amplicons were treated with shrimp alkaline phosphatase and exonuclease I [36], and DNA was quantified spectrophotometrically.

Amplicons were sequenced as part of a multi-species multi-sample Illumina HiSeq run [37], yielding partial contigs and also as pooled amplicons on a dedicated MiSeq run, yielding a complete mt genome. For the MiSeq run following agarose electrophoretic analysis, each amplicon was quantified fluorometrically using a Qubit 2.0 fluorometer (Invitrogen), pooled in equimolar ratios and prepared for paired-end sequencing on 1/10th Illumina MiSeq flow cell (v.3.0 chemistry; 2x 300 bp) using TruSeq nano DNA preparation kits [38]. Partial contigs from a previous mixed-sample HiSeq run (see [37] for details), including the present target amplicons, were used to map reference assemblies. Briefly, Trimmomatic [39] was used to trim the Illumina HiSeq reads. All complete nematode mt genomes available from the GenBank database were downloaded and protein-coding sequences extracted using Biopython [40]. Reads were interrogated using the GenBank protein sequences (each gene separately) employing usearch v.6.1 [41]. Matches were assembled into contigs (separately for each gene) using Mira v 4. [42]; these contigs were used as starters for mapping-extension using usearch to find reads, and Mira to enable assembly and further extension. The program CAP3 [43] was used to join contigs together. MiSeq reads were trimmed using the program Geneious (v.6.1.8, Biomatters) from both ends of each read, allowing a maximum of one ambiguous base and an error probability limit of 0.05, thus maximising the sequence length, whilst minimising the overall error to no more than 1 uncalled base. Previously, assembled contigs (above) were used to seed a high stringency mapping assembly in Geneious; one iteration at no mismatch, with no gaps allowed. Once matched to *P. univalens*, contigs were extended for a further 25 iterations using default (low sensitivity) assembly options, with no gaps allowed, minimum read overlap of 25 bp, minimum overlap identity of 90% and maximum mismatches set to 2% per read, to allow for assembly errors in the reference contigs. Resultant contigs were examined for overlapping regions and assembled into a complete mt genome. The mt genome (GenBank accession no. KM067271) was annotated using an established bioinformatic pipeline [22].

### Analyses of sequence data

The sequence was compared with the mt genome sequences of *A. suum* using the program Clustal X [44], and the circular map was drawn using the program MacVector v.9.5 (http://www.macvector.com/index.html). Amino acid sequences, translation initiation and termination codons, codon usage and transfer RNA (tRNA or *trn*) genes and non-coding regions were predicted using established approaches [22]. The structure and organisation of the mt genome of *P. univalens* was then compared with those of other ascaridoid nematodes *A. suum* (NC_001327); *A. lumbricoides* (NC_016198); *As. columbae* (NC_021643); *As. galli* (NC_021642); *An. simplex* (KC965056); *B. procyonis* (NC_016200); *B. transfuga* (NC_015924); *B. ailuri* (NC_015925); *B. schroederi* (NC_015927); *C. osculatum* (KC965057); *C. rudolphii* B (NC_014870); *Cu. robustus* (NC_016128); *T. canis* (NC_010690); *T. cati* (NC_010773); *T. malaysiensis* (NC_010527); *To. leonina* (NC_023504) [23–33].

### Phylogenetic analysis of concatenated amino acid sequence datasets

For phylogenetic analysis, amino acid sequences conceptually translated from individual protein-encoding genes of *P. univalens* and 16 reference mt genomes (Table 1) were aligned using MUSCLE, ensuring accurate alignment of homologous characters. Finally, all aligned blocks of sequences were concatenated and the entire alignment verified again by eye. These data were then subjected to phylogenetic analysis using Bayesian inference (BI). BI analysis was conducted using MrBayes v.3.2.2 [45] with a mixed amino acid substitution model [46] using four rate categories approximating a Γ distribution, four chains and 200,000 generations, sampling every 100th generation; the first 200 generations were removed from the analysis as burn-in.

**Table 1:** **Details of the mitochondrial genome sequence determined in this study and reference sequences used for analyses**

Species	Accession No.	Mt genome size	Host and geographical origins	Reference
*Parascaris univalens*	KM067271	13920	Horse (*Equus caballus*), Switzerland	This study
*Anisakis simplex (s.s.)*	KC965056	13926	Fish (*Clupea harengus*), Poland	[32]
*Ascaridia columbae*	NC_021643	13931	Pigeon (*Columba livia*), China	[30]
*Ascaridia galli*	NC_021642	13977	Chicken (*Gallus gallus*), China	[30]
*Ascaris lumbricoides*	HQ704900	14303	Human (*Homo sapiens*)*,* China	[33]
*Ascaris suum*	HQ704901	14311	Pig (*Sus scrofa*)*,* China	[33]
*Contracaecum osculatum*	KC965057	13823	Fish, (*Gadus morhua*)*,* Poland	[32]
*Contracaecum rudolphi* B	FJ905109	14022	Cormorant (*Phalacrocorax carbo*), China	[29]
*Cucullanus robustus*	NC_016128	13972	Conger eel (*Conger myriaster*)*,* Korea	[26]
*Baylisascaris ailuri*	NC_015925	14657	Red panda (*Ailurus fulgens*), China	[27]
*Baylisascaris procyonis*	JF951366	14781	Raccoon (*Procyon lotor*), China	[28]
*Baylisascaris schroederi*	NC_015927	14778	Giant panda (*Ailuropoda melanoleuca*), China	[27]
*Baylisascaris transfuga*	NC_015924	14898	Polar bear (*Ursus maritimus*), China	[27]
*Toxocara canis*	NC_010690	14322	Dog (*Canis lupus*)*,* China	[25]
*Toxocara cati*	NC_010773	14029	Cat (*Felis cati*)*,* China	[25]
*Toxocara malaysiensis*	NC_010527	14266	Cat (*Felis cati*)*,* China	[25]
*Toxascaris leonina*	NC_023504	14310	Dog (*Canis lupus*), Australia	[31]

## Results and discussion

### Features and organisation of the mt genome

**Figure 1:**
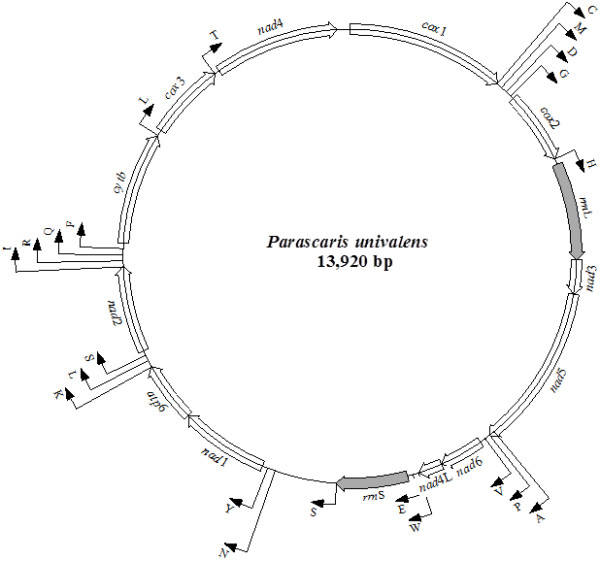
**Schematic representation of the circular mt genome of** ***Parascaris univalens.*** All 12 protein-coding genes and the large and small subunits of the rRNA genes are indicated in italics. Each tRNA gene is identified by its one letter abbreviation. The direction of transcription is indicated by arrows.

The circular mt genome of *P. univalens* (Figure 1) was 13,920 bp in length (GenBank accession number KM067271) and contained 36 genes: 12 protein-coding genes (adenosine triphosphatase subunit 6 [*atp6*]*,* the cytochrome *c* oxidase subunits 1, 2 and 3 [*cox*1*-cox*3]*,* cytochrome *b* (*cyt*b) and the nicotinamide dehydrogenase subunits 1–6 [*nad*1*-nad*6 and *nad*4L]), 22 tRNA genes (two coding for leucine and two coding for serine) and the small [*rrn*S] and large [*rrn*L] subunits of rRNA. Each protein-coding gene had an open reading frame (ORF), and all genes were located on the same strand and transcribed in the same direction (5′ to 3′) (Figure 1), consistent with the mt genomes of other secernentean nematodes characterised to date [20–22]. The gene arrangement (GA) for the mt genome of *P. univalens* was consistent with GA2 [47]. This gene arrangement has been reported previously for other ascaridoids; also, like other members of this nematode group, the AT-rich region for *P. univalens* was located between *rrn*S and *nad*1, flanked (5′) by the genes *trn*S (UCN) and (3′) by *trn*N and *trn*Y (Figure 1) [20].

### Nucleotide contents and codon usage

The coding strand of the mt genome sequence of *P. univalens* consisted of 19.1% A, 7.5% C, 22.12% G and 51.2% T (Table 2). Though AT-rich (78.1% AT), the sequence had a slightly lower AT content than has been reported for other nematode species (~70-80%; [20]); the AT-rich region was 828 bp in length (Table 2). In the protein-coding genes, the AT-contents varied from 65.5% (*cox*1) to 76.0% (*nad*4L), with the overall ranking (increasing richness) of *cox*1, *cox*2, *cox*3, *cyt*b, *nad*3, *nad*1, *atp*6, *nad*5, *nad*4, *nad*6*, nad*2, followed by *nad*4L. To date, studies of secernentean nematodes [20, 21] have shown that the cytochrome *c* oxidase genes tend to have the lowest AT-contents. Although the overall AT-content of the mt genome sequence of *P. univalens* was ~0.65-2.25% less than that of other ascaridoids studied to date [24, 25, 27, 28], there was no appreciable impact on the relative amino acid codon usage in the protein-coding genes. As has been reported for other secernentean nematodes [20, 21], the usage in the protein-coding genes favoured codons with many A or T residues (e.g., 14.1% were TTT [phenylalanine]) over those with many C or G residues (e.g., none were CGA [arginine]).

**Table 2:** **Nucleotide composition** ^**1**^ **(%) for the entire or regions/genes of the mitochondrial genome of** ***Parascaris univalens***

Mitochondrial gene/region	Length (bp)	A	C	G	T	AT
*atp*6	604	17.7	7.1	22.7	52.5	70.2
*cox*1	1,570	18.9	10.1	24.34	46.6	65.5
*cox*2	724	21.0	8.6	25.7	44.7	65.7
*cox*3	772	18.1	9.5	21.4	51.0	69.1
*cyt*b	1,108	19.0	9.0	21.8	50.1	69.1
*nad*1	880	19.3	8.3	21.9	50.5	69.8
*nad*2	859	19.3	5.7	19.8	55.2	74.5
*nad*3	340	17.1	4.1	26.2	52.6	69.7
*nad*4	1,240	18.9	10.0	19.0	52.1	71.0
*nad*4L	238	21.8	2.9	21.0	54.2	76.0
*nad*5	1,540	19.7	7.3	22.3	50.5	70.2
*nad*6	439	18.0	7.7	19.6	54.7	72.7
*rrn*L	961	26.4	6.3	19.5	47.9	74.3
*rrn*S	702	30.5	9.5	20.8	39.2	69.7
AT-rich	828	38.6	9.9	11.8	39.5	78.1
Genome	13,920	21.7	8.0	21.4	48.9	70.6

^1^Lengths and A + T contents (%) of the sequences of the 12 protein-coding genes, the large and small ribosomal RNA genes, the AT-rich region and of the entire mitochondrial genome of *P. univalens.*

All but the two serine tRNAs (AGN and UCN) had a predicted secondary structure containing a DHU arm and loop and a TV-replacement loop instead of the TψC arm and loop. As reported previously for secernentean nematodes [20], the two serine tRNAs each contained the TψC arm and loop but lacked the DHU arm and loop. The *rrn*L and *rrn*S genes were 961 and 702 bp in length. The AT-content of the sequences of *rrn*L, *rrn*S and the AT-rich (“control”) region were 74.3%, 69.7% and 78.1%, respectively. The relatively low AT-richness exhibited in the mt genome of *P. univalens* was pronounced for the rRNA genes. The AT-content of the *rrn*L sequence was 2.5% less compared with, for example, *A. suum* (76.8%) [23]. The AT-content of the *rrn*S sequence of *P. univalens* was 2.3% less than that reported for *A. suum* (71.9%) [23].

### Comparative analysis with other ascaridoids

Pairwise comparisons were made among the amino acid sequences inferred from individual protein-coding genes and the nucleotide sequences of the rRNA genes in the *P. univalens* mt genome with those representing 16 other ascaridoid nematodes (Table 3). The amino acid sequence similarities in individual inferred proteins ranged from 76.8% (CYTB) to 96% (COX3) between *P. univalens* and *A. suum*, and from 76.7% (NAD2) to 92% (COX2) between *P. univalens* and *An. simplex*. The amino acid sequence similarities between *P. univalens* and individual species of ascaridoids included here (*A. suum*, *A. lumbricoides*, *Ascaridia columbae*, *As. galli*, *B. procyonis*, *B. transfuga, B. ailuri, B. schroederi, An. simplex*, *C. osculatum, C. rudolphii* B, *Cu. robustus T. canis*, *T. cati, T. malaysiensis* and *To. leonina*; cf. Table 1) ranged from 41.8% (ATP6) to 97.5% (COX2), respectively. The nucleotide sequence similarities (Table 3) in *rrn*S and *rrn*L were 60.4-84.5% and 54.6-84.9% between *P. univalens* and other ascaridoids, respectively (Table 3). Additional file 1: Table S1 lists initiation and termination codons for protein-coding genes as well as the lengths of the amino acid sequences encoded in *P. univalens* compared with other ascaridoids (cf. Table 1).

**Table 3:** **Pairwise comparison** ^**1**^ **of the amino acid (aa) sequences of the 12 protein-encoding mitochondrial genes and in the nucleotide sequence of each of the two ribosomal genes**

***aa/rRNA***	***Al***	***As***	***Asc***	***Asg***	***Ans***	***Ba***	***Bp***	***Bs***	***Bt***	***Co***	***Cr***	***Cur***	***Tcan***	***Tcat***	***Tm***	***Tol***
ATP6	83.42	84.42	50.75	48.74	78.89	84.42	84.92	82.91	83.92	77.89	79.39	58.8	81.91	80.4	80.9	81.91
COX1	93.54	92.97	92.59	92.78	91.44	93.73	92.97	93.92	93.92	92.40	92.5	85.25	84.41	83.65	86.69	90.11
COX2	91.18	91.18	81.51	84.03	92.02	91.18	91.18	91.60	91.60	91.18	92.2	77.49	89.08	89.50	97.48	97.48
COX3	90.00	96.00	77.00	79.00	91.00	88.00	90.00	90.00	90.00	91.00	89.8	69.41	81.00	90.00	90.00	96.00
CYTB	80.80	76.80	74.13	71.20	78.13	79.47	78.40	78.13	77.87	77.60	73.35	68.95	60.00	60.53	90.93	91.47
NAD1	89.00	94.00	71.00	73.00	88.00	88.00	84.00	84.00	88.00	88.00	85.86	70	84.00	86.00	84.00	94.00
NAD2	74.91	77.03	53.71	54.06	76.68	73.85	77.39	78.80	76.68	77.74	69.04	41.78	72.79	84.10	89.75	78.45
NAD3	88.29	86.49	64.86	66.67	86.49	86.49	89.19	88.29	87.39	86.49	81.08	64.54	85.59	88.29	88.29	88.29
NAD4	82.48	82.73	82.00	81.75	80.78	81.02	76.89	82.48	80.78	82.00	73.59	63.57	67.40	67.64	80.05	81.51
NAD4L	89.61	88.31	58.44	54.55	84.42	89.61	90.91	90.91	90.91	89.61	88.31	68.83	87.01	84.42	85.71	83.12
NAD5	83.00	82.00	66.00	66.00	80.00	84.00	85.00	85.00	83.00	83.00	74.19	62.62	80.00	90.00	92.00	82.00
NAD6	79.00	80.00	52.00	52.00	70.00	79.00	78.00	77.00	77.00	72.00	68.05	59.72	74.00	76.00	75.00	75.00
rrnL	84.91	84.79	54.66	60.46	71.40	78.04	78.88	78.25	77.73	69.00	69.3	59.6	72.51	70.99	72.36	78.85
*rrn*S	84.00	84.45	63.39	64.05	76.14	82.45	83.71	81.41	82.42	74.00	75.9	60.4	75.32	75.14	75.72	82.71

^1^Percentage of similarity in the amino acid sequences inferred from the 12 protein-coding genes and in the nucleotide sequence of each of the two ribosomal genes (*rrn*L and *rrn*S) upon pairwise comparison between *Parascaris univalens* and selected ascaridoid nematodes, including *Ascaris lumbricoides* (*Al*)*, Ascaris suum* (*As*)*, Ascaridia columbae* (*Asc*), *Ascaridia galli* (*Asg*), *Anisakis simplex* (*Ans*), *Baylisascaris ailuri* (*Ba*), *Baylisascaris procyonis* (*Bp*)*, Baylisascaris schroederi* (*Bs*)*, Baylisascaris transfuga* (*Bt*)*, Contracaecum osculatum* (*Co*)*, Contracaecum rudolphii* B (*Cr*)*, Cucullanus robustus* (*Cur*)*, Toxocara canis* (*Tcan*)*, Toxocara cati* (*Tcat*)*, Toxocara malaysiensis* (*Tmal*)*, Toxascaris leonina* (*Tol*).

**Figure 2:**
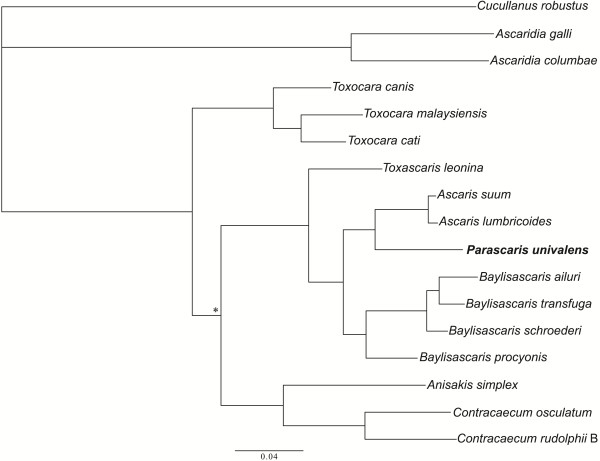
**Genetic relationship of** ***Parascaris univalens*** **with other ascaridoid nematodes.** Concatenated amino acid sequence data for all protein-encoding mitochondrial genes of *P. univalens* (bold) and other ascaridoids were subjected to Bayesian inference analysis. All nodes are supported by pp = 1.00, except that marked with an asterisk (pp = 0.98).

Subsequently, we undertook a phylogenetic analysis of the concatenated amino acid sequence data set representing *P. univalens* and reference sequences for selected ascaridoids (Figure 2). This analysis showed a robust estimate of interrelationships of *P. univalens* with selected ascaridoid nematodes, with each node strongly supported by a posterior probability (pp) value of 1.00 (see Figure 2). The analysis revealed that *P. univalens* grouped separately from *A. suum* and *A. lumbricoides* with absolute support (Figure 2). Within the monophyletic clade, *As. galli* and *As. columbae* grouped together to the exclusion of other species, which grouped together – here, *T. canis*, *T. cati* and *T. malaysiensis* grouped together, as did *B. ailuri*, *B. procyonis*, *B. schroederi* and *B. transfuga*, to the exclusion of *To. leonina*; *C. osculatum* and *C. rudolphii* B grouped together, to the exclusion of *An. simplex*.

### Significance and implications

There is considerable significance in the use of mt DNA markers for investigating the genetic make-up of *Parascaris* populations, particularly given that there are no morphological features that allow the specific identification of most developmental stages. In nematodes, mt DNA is proposed to be maternally inherited, and is usually more variable in sequence within a species than nuclear ribosomal DNA [19, 20]. The complete mt genome of *P. univalens* characterised here provides a likely foundation for assessing the extent of genetic variation within and between *P. univalens* and *P. equorum* populations, and might allow the definition of markers for specific PCR-based identification/differentiation of these nematodes at any stage of development.

Oligonucleotide primers could be selectively designed to conserved regions flanking “variable tracts” in the mt genome considered to be most informative following mt sequencing from a relatively small number of individuals identified as *P. univalens* and *P. equorum* by cytological analysis (cf. [15]). Using such primers, PCR-coupled single-strand conformation polymorphism (SSCP) analysis [34] might be employed to screen large numbers of *Parascaris* individuals representing different populations, and samples representing the spectrum of haplotypic variability could then be selected for subsequent sequencing and analyses. As the two internal transcribed spacers (ITS-1 and ITS-2) of nuclear ribosomal DNA usually provide species-level identification of closely and distantly related ascaridoids [19, 48–50], comparative genetic analyses using these spacers would also be informative. Such approaches have been applied, for example, to study the genetic make-up of the *Ascaris* populations in humans and pigs in six provinces in China [51, 52]; like *A. suum* and *A. lumbricoides*, *P. univalens* and *P. equorum* are recognised to be very closely related taxa [53, 54], and based on early observations, there is some evidence that *P. univalens* and *P. equorum* might hybridise but produce infertile offspring [15]. Having available reliable markers and molecular tools might not only enable studies of the biology and epidemiology of these parasites, but also investigations into anthelmintic resistance in *P. univalens* and *P. equorum*, in combination with conventional faecal egg count reduction testing (FECRT) [55, 56], as well as single nucleotide polymorphism (SNP) analyses of genes inferred to be involved in such resistance [57, 58].

## Conclusions

The mt genome of *P. univalens* provides a rich source of genetic markers for future studies of the population genetics and epidemiology of *Parascaris* in horses. It sets the scene particularly for large-scale genetic studies of *Parascaris* from domestic and wild horses around the world. This focus is significant, given that there is no published information on the prevalence and distribution of *P. univalens* in domestic and wild horses or anthelmintic resistance in this species.

## Electronic supplementary material

Additional file 1: Table S1: Comparison of the mt genome of *Parascaris univalens* with those of other ascaridoid nematodes. (DOCX 39 KB)

## Abbreviations

*atp*6: adenosine triphosphatase subunit 6
BI: Bayesian inference
*cox*: cytochrome *c* subunit
*cyt*b: cytochrome *b* subunit
ITS-2: second internal transcribed spacer
mt: mitochondrial
*nad*: nicotinamide dehydrogenase subunit
ORF: open reading frame
rDNA: nuclear ribosomal DNA
*rrn*L: large subunit of the ribosomal gene
*rrn*S: small subunit of the ribosomal gene
tRNA: transfer RNA.
