# Supplementary material for: The mitochondrial genome of Parascaris univalens - implications for a “forgotten” parasite
Source: Parasit Vectors. 2014 Sep 4;7:428. doi: 10.1186/1756-3305-7-428 (PMC4262126; doi:10.1186/1756-3305-7-428)
Supplement: Supplementary file 1 — Additional file 1: Table S1: Comparison of the mt genome of Parascaris univalens with those of other ascaridoid nematodes. (DOCX 39 KB) [file 13071_2014_1604_MOESM1_ESM.docx]

**Supplementary Table 1 Comparison^1^ of the mt genome of *Parascaris univalens* with those of other ascaridoid nematodes.**

| Gene | Parascaris univalens | | Anisakis simplex | | Ascaridia columbae | | Ascaridia galli | | Ascaris lumbricoides | |
| --- | --- | --- | --- | --- | --- | --- | --- | --- | --- | --- |
|  | Length (aa) | Ini/Ter codons | Length (aa) | Ini/Ter codons | Length (aa) | Ini/Ter codons | Length (aa) | Ini/Ter codons | Length (aa) | Ini/Ter codons |
|  |  |  |  |  |  |  |  |  |  |  |
| *cox*1 | 521 | TTG/TAG | 525 | TTG/T | 520 | ATG/TAA | 520 | ATG/TAA | 525 | ATT/TAG |
| *trn*C |  |  |  |  |  |  |  |  |  |  |
| *trn*M |  |  |  |  |  |  |  |  |  |  |
| *trn*D |  |  |  |  |  |  |  |  |  |  |
| *trn*G |  |  |  |  |  |  |  |  |  |  |
| *cox*2 | 232 | TTG/TAG | 232 | TTG/TAG | 231 | TTG/TAG | 231 | TTG/TAG | 232 | TTG/TAG |
| *trn*H |  |  |  |  |  |  |  |  |  |  |
| *rrn*L |  |  |  |  |  |  |  |  |  |  |
| *nad*3 | 111 | TTG/TAG | 111 | TTG/TAG | 111 | ATA/TAA | 111 | TTG/TAA | 111 | TTG/TAG |
| *nad*5 | 528 | ATT/T | 527 | ATT/T | 526 | ATA/TAG | 526 | ATA/TAA | 528 | ATT/T |
| *trn*A |  |  |  |  |  |  |  |  |  |  |
| *trn*P |  |  |  |  |  |  |  |  |  |  |
| *trn*V |  |  |  |  |  |  |  |  |  |  |
| *nad*6 | 144 | ATG/TAG | 144 | TTG/TAA | 144 | TTG/TAA | 144 | TTT/TAA | 144 | TTG/TAG |
| *nad*4L | 77 | ATT/TAA | 77 | ATT/T | 72 | GTT/T | 72 | ATT/TAA | 77 | ATT/TAG |
| *trn*W |  |  |  |  |  |  |  |  |  |  |
| *trn*E |  |  |  |  |  |  |  |  |  |  |
| *rrn*S |  |  |  |  |  |  |  |  |  |  |
| *trn*S (UCN) |  |  |  |  |  |  |  |  |  |  |
| *trn*N |  |  |  |  |  |  |  |  |  |  |
| *trn*Y |  |  |  |  |  |  |  |  |  |  |
| *nad*1 | 290 | TTG/TAG | 290 | ATA-TAA | 291 | TTG/TAA | 291 | TTG/TAA | 290 | TTG/TAG |
| *atp*6 | 199 | ATT/TAA | 199 | TTG-TAA | 198 | ATA/TAA | 198 | ATA/TAA | 199 | ATT/TAG |
| *trn*K |  |  |  |  |  |  |  |  |  |  |
| *trn*L (UUR) |  |  |  |  |  |  |  |  |  |  |
| *trn*S (AGN) |  |  |  |  |  |  |  |  |  |  |
| *nad*2 | 281 | TTG/T | 281 | TTG/TAA | 280 | TTG/TAA | 280 | TTG/TAA | 281 | TTG/T |
| *trn*I |  |  |  |  |  |  |  |  |  |  |
| *trn*R |  |  |  |  |  |  |  |  |  |  |
| *trn*Q |  |  |  |  |  |  |  |  |  |  |
| *trn*F |  |  |  |  |  |  |  |  |  |  |
| *cob* | 364 | GTT/TAG | 366 | ATG/T | 366 | GTT/TAA | 367 | ATG/TAA | 365 | ATT/TAA |
| *trn*L (CUN) |  |  |  |  |  |  |  |  |  |  |
| *cox*3 | 255 | GTT/TAG | 255 | TTG/T | 248 | TTG/TAA | 248 | TTG/TAG | 255 | GTT/TAG |
| *trn*T |  |  |  |  |  |  |  |  |  |  |
| *nad*4 | 409 | TTG/TAA | 409 | TTG/TAA | 411 | ATA/TAG | 411 | GTG/TAG | 409 | TTG/TAA |

^1^Characteristics of *Parascaris univalens* mt genome, including initiation and termination codons for protein-coding genes as well as the lengths of their predicted amino acid sequences, compared with those of selected ascaridoid nematodes (cf. Table 1).

| Gene | Ascaris suum | | Contracaecum osculatum | | Contracaecum rudolphii B | | Cucullanus robustus | | Baylisascaris ailuri | |
| --- | --- | --- | --- | --- | --- | --- | --- | --- | --- | --- |
|  | Length (aa) | Ini/Ter codons | Length (aa) | Ini/Ter codons | Length (aa) | Ini/Ter codons | Length (aa) | Ini/Ter codons | Length (aa) | Ini/Ter codons |
| *cox*1 | 525 | ATT/T | 525 | TTG/T | 525 | TTG/T | 525 | TTG/TAG | 524 | TTG/TAA |
| *trn*C |  |  |  |  |  |  |  |  |  |  |
| *trn*M |  |  |  |  |  |  |  |  |  |  |
| *trn*D |  |  |  |  |  |  |  |  |  |  |
| *trn*G |  |  |  |  |  |  |  |  |  |  |
| *cox*2 | 232 | TTG/TAG | 231 | TTG/TAA | 231 | TTG/TAA | 231 | TTG/TAA | 232 | TTG/TAG |
| *trn*H |  |  |  |  |  |  |  |  |  |  |
| *rrn*L |  |  |  |  |  |  |  |  |  |  |
| *nad*3 | 111 | TTG/TAG | 111 | TTG/TAA | 111 | TTG/TAA | 110 | TTG/TAA | 111 | TTG/TAG |
| *nad*5 | 528 | ATT/T | 527 | ATT/T | 527 | ATT/T | 527 | ATT/TAA | 528 | ATT/T |
| *trn*A |  |  |  |  |  |  |  |  |  |  |
| *trn*P |  |  |  |  |  |  |  |  |  |  |
| *trn*V |  |  |  |  |  |  |  |  |  |  |
| *nad*6 | 144 | TTG/TAG | 144 | TTG/TAA | 144 | TTG/TAA | 144 | ATT/TAA | 144 | TTG/TAG |
| *nad*4L | 77 | ATT/TAG | 77 | ATT/TAG | 77 | ATT/TAG | 77 | ATT/TAA | 77 | ATT/TAG |
| *trn*W |  |  |  |  |  |  |  |  |  |  |
| *trn*E |  |  |  |  |  |  |  |  |  |  |
| *rrn*S |  |  |  |  |  |  |  |  |  |  |
| *trn*S (UCN) |  |  |  |  |  |  |  |  |  |  |
| *trn*N |  |  |  |  |  |  |  |  |  |  |
| *trn*Y |  |  |  |  |  |  |  |  |  |  |
| *nad*1 | 290 | TTG/TAG | 290 | TTG/TAA | 290 | TTG/TAA | 290 | TTG/TAG | 290 | TTG/TAG |
| *atp*6 | 199 | ATT/TAG | 199 | ATT/TAA | 199 | ATT/TAG | 199 | ATT/TAA | 199 | ATT/TAG |
| *trn*K |  |  |  |  |  |  |  |  |  |  |
| *trn*L (UUR) |  |  |  |  |  |  |  |  |  |  |
| *trn*S (AGN) |  |  |  |  |  |  |  |  |  |  |
| *nad*2 | 281 | TTG/T | 281 | ATT/TAG | 281 | TTG/TAA | 280 | TTG/TAA | 281 | TTG/TA |
| *trn*I |  |  |  |  |  |  |  |  |  |  |
| *trn*R |  |  |  |  |  |  |  |  |  |  |
| *trn*Q |  |  |  |  |  |  |  |  |  |  |
| *trn*F |  |  |  |  |  |  |  |  |  |  |
| *cob* | 365 | ATT/TAG | 368 | TTG/TAA | 368 | TTG/TAG | 366 | ATT/T | 368 | GTG/TAA |
| *trn*L (CUN) |  |  |  |  |  |  |  |  |  |  |
| *cox*3 | 255 | GTT/TAA | 255 | ATT/T | 255 | TTG/T | 255 | TTG/T | 255 | ATG/TAG |
| *trn*T |  |  |  |  |  |  |  |  |  |  |
| *nad*4 | 409 | TTG/TAA | 409 | TTG/TAG | 409 | TTG/TAA | 409 | TTG/TAA | 409 | TTG/TAG |

| Gene | Baylisascaris procyonis | | Baylisascaris schroederi | | Baylisascaris transfuga | | Toxocara canis | | |
| --- | --- | --- | --- | --- | --- | --- | --- | --- | --- |
|  | Length (aa) | Ini/Ter codons | Length (aa) | Ini/Ter codons | Length (aa) | Ini/Ter codons | Length (aa) | Ini/Ter codons | |
|  |  |  |  |  |  |  |  |  | |
| cox1 | 525 | TTG/TAG | 525 | TTG/TAA | | 525 | TTG/TAA | 526 | TTG/TAG |
| *trn*C |  |  |  |  | |  |  |  |  |
| *trn*M |  |  |  |  | |  |  |  |  |
| *trn*D |  |  |  |  | |  |  |  |  |
| *trn*G |  |  |  |  | |  |  |  |  |
| *cox*2 | 232 | TTG/TAG | 232 | TTG/TAG | | 232 | TTG/TAG | 238 | GTT/TAG |
| *trn*H |  |  |  |  | |  |  |  |  |
| *rrn*L |  |  |  |  | |  |  |  |  |
| *nad*3 | 111 | TTG/TAA | 111 | TTG/TAG | | 111 | TTG/TAG | 112 | TTG/TAG |
| *nad*5 | 528 | ATT/T | 528 | ATT/T | | 528 | ATT/T | 528 | ATA/T |
| *trn*A |  |  |  |  | |  |  |  |  |
| *trn*P |  |  |  |  | |  |  |  |  |
| *trn*V |  |  |  |  | |  |  |  |  |
| *nad*6 | 144 | TTG/TAG | 144 | TTG/TAG | | 144 | TTG/TAG | 145 | TTG/TA |
| *nad*4L | 77 | ATT/TAA | 77 | ATT/TAG | | 77 | ATT/TAG | 78 | ATT/T |
| *trn*W |  |  |  |  | |  |  |  |  |
| *trn*E |  |  |  |  | |  |  |  |  |
| *rrn*S |  |  |  |  | |  |  |  |  |
| *trn*S (UCN) |  |  |  |  | |  |  |  |  |
| *trn*N |  |  |  |  | |  |  |  |  |
| *trn*Y |  |  |  |  | |  |  |  |  |
| *nad*1 | 290 | TTG/TAG | 290 | TTG/TAG | | 290 | TTG/TAG | 291 | TTG/TAG |
| *atp*6 | 199 | ATA/TAG | 199 | ATT/TAA | | 199 | ATT/TAG | 200 | ATT/T |
| *trn*K |  |  |  |  | |  |  |  |  |
| *trn*L (UUR) |  |  |  |  | |  |  |  |  |
| *trn*S (AGN) |  |  |  |  | |  |  |  |  |
| *nad*2 | 281 | GTG/T | 281 | TTG/TA | | 281 | TTG/TA | 282 | ATT/T |
| *trn*I |  |  |  |  | |  |  |  |  |
| *trn*R |  |  |  |  | |  |  |  |  |
| *trn*Q |  |  |  |  | |  |  |  |  |
| *trn*F |  |  |  |  | |  |  |  |  |
| *cob* | 368 | GTG/TAG | 368 | GTG/TAG | | 368 | GTG/TAG | 369 | GTG/TAG |
| *trn*L (CUN) |  |  |  |  | |  |  |  |  |
| *cox*3 | 255 | GTG/TAG | 255 | ATG/TAG | | 255 | ATG/TAG | 256 | ATG/TAG |
| *trn*T |  |  |  |  | |  |  |  |  |
| *nad*4 | 409 | TTG/TAG | 409 | TTG/TAG | | 409 | TTG/TAG | 410 | ATA/TAG |

| Gene | Toxocara cati | | Toxocara malaysiensis | | Toxascaris leonina | |
| --- | --- | --- | --- | --- | --- | --- |
|  | Length (aa) | Ini/Ter codons | Length (aa) | Ini/Ter codons | Length (aa) | Ini/Ter codons |
|  |  |  |  |  |  |  |
| *cox*1 | 526 | TTG/TAG | 527 | TTG/TAG | 525 | TTG/TAG |
| *trn*C |  |  |  |  |  |  |
| *trn*M |  |  |  |  |  |  |
| *trn*D |  |  |  |  |  |  |
| *trn*G |  |  |  |  |  |  |
| *cox*2 | 237 | GTT/TAA | 237 | GTA/TAG | 232 | TTG/T |
| *trn*H |  |  |  |  |  |  |
| *rrn*L |  |  |  |  |  |  |
| *nad*3 | 112 | TTG/TAG | 112 | TTG/TAG | 111 | TTG/TAG |
| *nad*5 | 528 | ATA/T | 528 | ATG/T | 527 | ATT/T |
| *trn*A |  |  |  |  |  |  |
| *trn*P |  |  |  |  |  |  |
| *trn*V |  |  |  |  |  |  |
| *nad*6 | 145 | TTG/TA | 145 | TTG/TA | 144 | TTG/TAG |
| *nad*4L | 78 | ATT/T | 78 | ATT/T | 77 | ATT/TAG |
| *trn*W |  |  |  |  |  |  |
| *trn*E |  |  |  |  |  |  |
| *rrn*S |  |  |  |  |  |  |
| *trn*S (UCN) |  |  |  |  |  |  |
| *trn*N |  |  |  |  |  |  |
| *trn*Y |  |  |  |  |  |  |
| *nad*1 | 291 | TTG/TAA | 291 | TTG/TAG | 290 | TTG/TAG |
| *atp*6 | 200 | ATT/T | 200 | ATT/T | 199 | ATT/TAG |
| *trn*K |  |  |  |  |  |  |
| *trn*L (UUR) |  |  |  |  |  |  |
| *trn*S (AGN) |  |  |  |  |  |  |
| *nad*2 | 282 | GTT/T | 282 | ATT/T | 281 | GTG/T |
| *trn*I |  |  |  |  |  |  |
| *trn*R |  |  |  |  |  |  |
| *trn*Q |  |  |  |  |  |  |
| *trn*F |  |  |  |  |  |  |
| *cob* | 369 | ATG/TAA | 369 | GTG/TAG | 364 | TTG/TAA |
| *trn*L (CUN) |  |  |  |  |  |  |
| *cox*3 | 256 | ATG/TAA | 256 | GTG/TAG | 255 | GTT/TAG |
| *trn*T |  |  |  |  |  |  |
| *nad*4 | 410 | ATA/TAG | 410 | ATA/TAG | 409 | TTG/TAG |
